# Percutaneous stent implantation for occluded central shunts in adults: A case report and review of current evidence

**DOI:** 10.3389/fcvm.2022.1032974

**Published:** 2022-11-21

**Authors:** Yaser Jenab, Malihe Rezaee, Kaveh Hosseini, Homa Ghaderian, Raymond N. Haddad, Ali N. Zaidi

**Affiliations:** ^1^Tehran Heart Center, Cardiovascular Diseases Research Institute, Tehran University of Medical Sciences, Tehran, Iran; ^2^School of Medicine, Shahid Beheshti University of Medical Sciences, Tehran, Iran; ^3^M3C-Necker, Hôpital Universitaire Necker-Enfants Malades, Assistance Publique-Hôpitaux de Paris (AP-HP), Paris, France; ^4^Mount Sinai Adult Congenital Heart Disease Center, Icahn School of Medicine, New York, NY, United States

**Keywords:** aortopulmonary shunt, systemic-to-pulmonary shunt, stent, percutaneous intervention, central shunt

## Abstract

**Background:**

Patients with cyanotic complex congenital heart defects (CHDs) commonly undergo palliation with interposition of systemic-to-pulmonary shunts (SPSs). These palliative shunts are rarely found in adults with CHDs and can be complicated with progressive obstruction or total occlusion during follow-up. The best treatment option for shunt re-permeabilization is challenging and case-oriented because most patients are high risk candidates for redo surgeries. We aimed to review the current evidence on percutaneous stent implantation to treat failed SPSs.

**Methods:**

We performed a comprehensive literature review on percutaneous stent implantation to treat failed and occluded SPSs. We also reported the case of a 33-year-old man with cyanotic CHD and an occluded central aorto-pulmonary shunt, who was successfully treated with percutaneous balloon dilatation and subsequently stent implantation at our institution.

**Result:**

We identified and included 31 articles reporting on 150 patients and 165 stent implantations in failed SPSs. The age of patients at the time of stent implantation ranged from 6 days to 47 years. The time between the surgical shunt creation and transcatheter intervention ranged from 1 day to 17 years. Overall, 161/165 (97.5%) stent implantations were successful. The most common clinical presentation was cyanosis and decreased atrial oxygen saturations and the indication for stent implantation was shunt obstruction and stenosis.

**Conclusion:**

This review highlights the benefits of endovascular stenting to permeabilize failed SPSs in children and adults with complex CHD who are classified as poor candidates for re-surgical repair.

## Graphical Abstract

Successful percutaneous stent implantation for re-opening the occluded central aorto-pulmonary shunt in adults. 
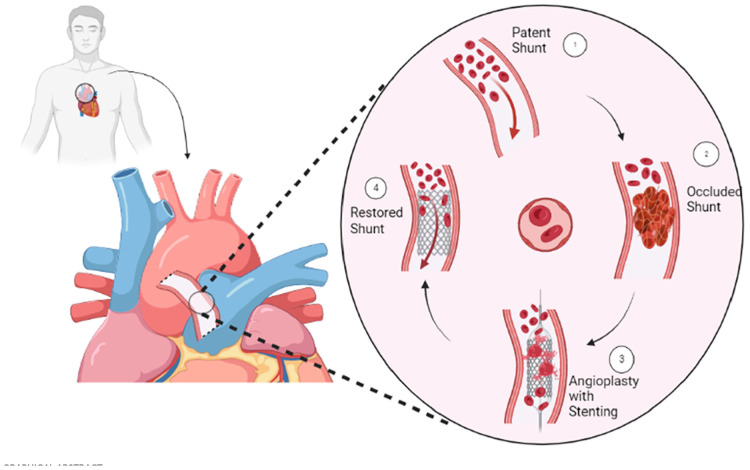

## Introduction

Despite advancements in corrective surgeries for congenital heart defects (CHDs), systemic-to-pulmonary arterial shunts (SPSs) remain the most common surgical palliation in complex CHDs with inadequate pulmonary blood flow (1). Various types of surgical shunts are currently proposed including the classical or modified Blalock-Taussig shunt (BTS), and the central aortopulmonary shunt (APS) (2–6). Central APS is preferred in patients with small pulmonary arteries (5, 7–9). Surgical SPSs usually provide short-term palliation, yet they may be associated with early and late complications leading to significant morbidity and mortality (10–12). Regardless of the type of shunt, short- and long-term failure could occur (13, 14). Shunt stenosis and occlusion have been frequently reported in infants and adults with complex cyanotic CHDs and are most commonly secondary to thrombosis, vascular distortion, suture line stricture, and intimal proliferation (15–17). Shunt failure can develop suddenly or gradually leading to restriction of pulmonary perfusion with subsequent respiratory distress, severe hypoxia, and cyanosis (18). In these cases, surgical repair is recognized as the gold standard therapy, yet most of these patients are classified as high-risk candidates for redo-sternotomies (19, 20). Therefore, transcatheter repair of occluded SPSs is an appealing option in neonates and infants (21, 22). On the other side, palliative SPSs in adults are rare but shunt obstruction is common and progressive, leading to hypoxemia and exertional dyspnea (23, 24). Likewise, transcatheter correction is interesting in adults but the intervention might be complicated by the complex anatomy and the shunt calcification (25–27). To our knowledge, the evidence on stent implantation to re-open occluded APS in adults is scarce. Herein, we report an interesting case of percutaneous permeabilization of an occluded central APS in an adult patient and discuss a comprehensive review of the literature on this topic.

## Methods

We performed a literature search in Web of Science, PubMed, Scopus, and Google Scholar databases from the beginning to April 2022, without any limitation. The keywords used the title and abstract for the search included transcatheter implantation of stents in the SPS, BTS, modified BTS, APS, or central shunt. A total of 1,313 articles were compiled. Two reviewers independently screened the retrieved studies. After removing duplicate articles, the initial assessment was performed based on the relevance of the title and abstract of the articles. Subsequently, the full texts of the remaining articles were acquired and reviewed to be enrolled in the present review. Reference lists of the included articles were also searched for additional related manuscripts.

## Results

**FIGURE 1:**
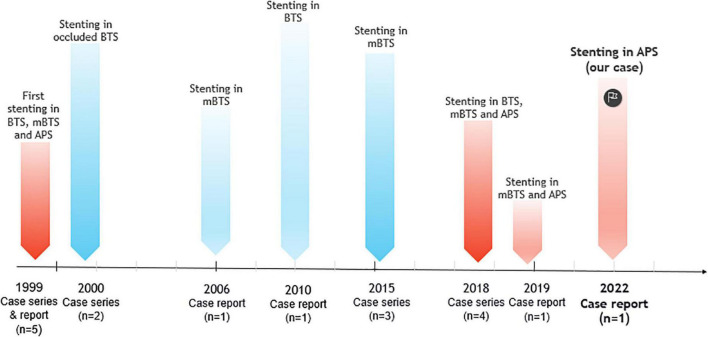
Timeline of endovascular stent implantation in systemic-to-pulmonary arterial shunts (SPSs) including, classical Blalock–Taussig shunt (BTS), modified Blalock–Taussig shunt (mBTS), and central aorto-pulmonary shunt (APS) in adult patients (≥18 years old).

We identified 31 articles reporting on 150 patients with failing SPSs and receiving 165 stents implantation. 108 (72%) patients had classical or modified BTS, 37 (24.7%) patients had APS, 3 (2%) patients had both BTS and APS, one patient had a left-sided SPS, and one patient had a right internal mammary artery (RIMA) to pulmonary artery shunt. Of three patients with both BTS and APS, two patients received stent insertion only in BTS and the other patient had stent implantation in both shunts. The age of shunt creation varied from 1 day to 28 years. The age at the time of stent implantation ranged from 6 days to 47 years. The time between the surgical shunt creation and transcatheter intervention ranged from 1 day to 17 years. The gender of patients was reported in 22 studies compromising 71 patients with 31 females and 40 males. Overall, 161/165 (97.5%) stent implantations in SPS were successful. Shunt stenosis and thrombosis were the most indications for endovascular stent implantation and patients mainly presented with cyanosis and decreased atrial oxygen saturation. Stent placement was done in three and two patients with APS and BTS, respectively, to downsize the internal lumen of the shunt due to pulmonary overflow; and in two patients to treat a pseudoaneurysm of BTS. One patient with a history of percutaneously treated stenotic shunt underwent after 5 years a stent implantation in a left-sided SPS after a bacterial abscess induced shunt obstruction. The included articles in this review are outlined in Table 1. The reported stent implantations in adults with SPSs from beginning until now are illustrated in Figure 1.

**TABLE 1:** Characteristics of the studies.

References	Year of publication	Type of study	Number of patients	Age at shunt creation	Sex	Congenital heart disease	Type of shunt	Age at stenting	Time from shunt to stent	Indication of stent implantation	Symptom before stenting	Outcome
Zahn et al. (45)	1997	Case report	1	8 days	NR	HLHS	mBTS	Neonate	NR	Shunt thrombosis and stenosis	Progressively cyanosis Dropped SpO2 Hypotension	Successful
Peuster et al. (98)	1998	Case series	2	10 days	NR	TA	BTS	15 days	5 days	Thrombotic shunt occlusion	Hypoxemia Dropped SpO2	Successful
				4 weeks	NR	TOF with hypoplastic pulmonary arteries	BTS	6 weeks	2 weeks			
Alcíbar et al. (47) Not English (Based on abstract)	1999	Case report	2	NR	NR	Complex cyanotic CHD	Central APS	1 and 13 months	NR	Shunt occluded	Severe hypoxemia	Successful
Alcibar et al. (46) Full text not found (Based on abstract)	1999	Case report	1	NR	NR	TA	Central APS	5.5 W	NR	Severe postoperative stenosis	Severe hypoxia	Successful
Bader et al. (48)	1999	Case series	4	5 – 29 years	F	Complex cyanotic CHD	1 BTS 2 mBTS 1 central APS	23–32 years	2–21 years	Long segment stenosis of shunts	Increasing cyanosis Decrease SpO2	Successful
Benito Bartolomé et al. (23) In Spanish (Based on abstract)	1999	Case report	1	In infancy	F	PA/VSD	Classic BTS	26 years	NR	Complete obstruction	Progressive cyanosis Dyspnea	Successful
El-Said et al. (53)	2000	Case series	2 (2 of 3 patients underwent stent in shunt)	28 years	F	PA/VSD	BTS	33 years	5 years	A discrete stenosis	Increasing cyanosis Dropped SpO2 Hypoxemia	Successful
				8 years	M	PA/VSD	mBTS	11 years	3 years	Severe shunt narrowing		
Lee et al. (29)	2001	Case series	13 (15 stent implantations)	1 day– 8 year	8 M 5 F	Complex cyanotic CHD	Two classic BTS 10 mBTS 1 central APS	14 days–12.5 years	Median: 1.4 year (1 day–8 year)	Shunt stenosis or occlusion	Low arterial SpO2 Change in the shunt murmur	Successful
Tomita et al. (66)	2002	Case report	1	1 month	M	PA	mBTS	3 year	2 year and 11 month	Acute obstruction following selective angiography	Decrease the SpO2	Successful
Moszura et al. (65) In polish (Based on abstract)	2004	Case report	1	NR	M	Complex cyanotic CHD	mBTS	5 year	NR	NR	NR	Successful
Kouatli et al. (52)	2005	Case report	1	15 days	F	Complex cyanotic CHD	Classical BTS	10 years	10 years	Severe stenosed shunt	Severe cyanosis Decreased SpO2 Severe limitation in physical activity	Successful
Maree and Walsh (49) Full text not found	2006	Case report	1	4 years	F	Complex cyanotic CHD	mBTS	21 years	17 years	Narrowed shunt with impaired blood flow	Declining exercise tolerance Presyncope Increasing cyanosis	successful
Krasemann and Qureshi (97)	2007	Case report	1	3 days	M	HLHS	mBTS	50 days	47 days	shunt stenosis	SPO2 dropped Quieter shunt murmur	Successful
Sreeram et al. (96)	2008	Case series	7	NR	NR	Complex cyanotic CHD	Six central APS 1 mBTS	6 days–7 months	NR	Acute shunt occlusion	Acute decreased SpO2	Successful
Kaestner et al. (71)	2008	Case series	3 (3 of 5 patients have SPS)	NR	NR	Complex cyanotic CHD	Two central APS 1 mBTS	12– 62 days	NR	Partial or complete occluded shunt	Decreased SpO2	Successful
Moszura et al. (34)	2010	Case series	3 (3 of 23 have stent implantations)	NR	M	Complex cyanotic CHD	mBTS	18 days	5 days	Occlusion or stenosis of shunts	Decreased SpO2	3 successful
								2 years	3 years			
								5 years	8 months			
Sanchez-Recalde et al. (50)	2010	Case report	1	BTS inserted in 4 years old Central APS inserted in 6 years old	F	PA/VSD	Classic BTS and central APS (Just stenting in BTS)	26 years	22 years	Complete obstruction of the BTS	Progressive cyanosis Dyspnea	Successful
								40 years	14 years	Pseudoaneurysm- with a dissection flap inside immediately proximal to the stent	In CT and CXR	Successful
Krasemann et al. (37)	2011	Case series	7	NR	6 M 1 F	Complex cyanotic CHD	Two Classical/5 modified BTS	25–218 days	2–137 days	Shunt stenosis or occlusion	Low SpO2 Tachypnoea	Seven successful
Lee and Chiu (38)	2012	Case report	1	1 week	M	Complex cyanotic CHD with PA	Bilateral mBTS	5.75 years	5 years	Severe stenosis of both BT shunts	Decrease SpO2 at rest and in sleep Cyanosis of lip Clubbing Quieter shunt murmur	Successful
McMahon et al. (99)	2013	Case report	1	4 months	M	Complex cyanotic CHD with PA	Central APS	16 months	12 months	Shunt stenosis	Decreased SpO2	Successful
Bonnet et al. (31)	2015	Case series	14 (14 of 28 have stent implantations)	0.01–18 years	M/F sex ratio = 1.15	PS/PA only or with another defects	mBTS	0.03–32 years with (18% of adults (>15 years)	NR	Shunt occlusion	Decrease SpO2 Change in murmur Dyspnea	13 successful 1 unsuccessful
Vaughn et al. (35)	2015	Case series	22 (25 stent implantation)	NR	9 F 13 M	Complex cyanotic CHD with PA	13 mBTS 7 central APS 1 RIMA to pulmonary artery shunt 1 both central and BTS	10 days–4 year	Median 1.9 m (4 days–3.8 years)	Shunt occlusion or narrowing	Progressive cyanosis Oxygen requirement Respiratory distress Increasing cyanosis Cardiovascular collapse	25 Successful
Fiszer et al. (78)	2016	Case report	1	7 years	F	Complex cyanotic CHD	BTS	16 years	9 years	Oversized shunt	Massive left sided pleural effusions with hemodynamic compromise	Successful
Baspinar et al. (86)	2016	Case report	2	3 years and 10 months	F	DOVR with PS	mBTS	4 years	2 months	Pseudoaneurysm of shunt	Intermittent massive pulmonary hemorrhage Decrease SpO2 Enlarged upper left mediastinum	Successful
				7 years	M	Complex cyanotic CHD	mBTS	7 years and 5 months	5 months	Pseudoaneurysm of shunt	Massive hemoptysis	Successful
Cools et al. (90)	2017	Case series	11 (11 of 19 patients, need stent)	0.2–16 months	NR	Complex cyanotic CHD	Central APS	2–20 months	Median: 3 month (0.9–4.4 months)	Shunt stenosis	Decrease SpO2	11 successful
Ligon et al. (20)	2017	Case series	34 patients (42 stent insertion)	NR	NR	Complex cyanotic CHD	BTS	8 - 1,634 days	NR	Stenotic shunt or occlusion	Cyanosis	39 of 42 stents (93%) successful 3 of 49 stents unsuccessful
Kasem et al. (19)	2018	Case series	5	NR	4 F 1 M	Complex cyanotic CHD with PA	1 Classical BTS 2 mBTS 2 Central APS	17–45 years	NR	Stenotic shunt or occlusion	Decrease SpO2 Progressive cyanosis	Five successful
Illner et al. (25)	2019	Case report	1	7 years	M	PA/VSD	mBTS and central APS (Stenting just in mBTS)	47 years	20 years	Occluded BT shunt	Progressive fatigue Limited walking distance Hypoxemia Decreased SPO2	Successful
Maschietto et al. (95)	2020	Case series	4	NR	NR	Complex cyanotic CHD	Three central APS 1 mBTS	0.7–5.7 years	median: 56 days (26 –160 days)	Oversized shunt	Signs or symptoms of pulmonary over circulation	Four successful
Gopalakrishnan et al. (36)	2020	Case report	1	7 months	M	DOVR/PS	BTS	7 months	10 days	Acute shunt thrombosis	Decrease SPO2 Shock Absence the Shunt murmur	Successful
Homma and Hayabuchi (27)	2020	Case report	1	1 month	F	Single ventricle/PA/arterial-pulmonary collateral arteries	left-sided systemic-to-pulmonary shunt	8 years	8 years	Shunt stenosis	Decrease SPO2 Hemodynamic instability	successful
								13 years	13 years	Shunt obstruction due to a Staphylococcus aureus abscess		

APS, aorto-pulmonary shunt; ARF, acute renal failure; CHD, congenital heart disease; DOVR, double-outlet right ventricle; HLHS, hypoplastic left heart syndrome; mBTS, modified Blalock-Taussig shunt; PA, pulmonary atresia; TA, tricuspid atresia; TOF, tetralogy of fallot; VAD, ventricular septal defect.

## Brief case presentation

**FIGURE 2:**
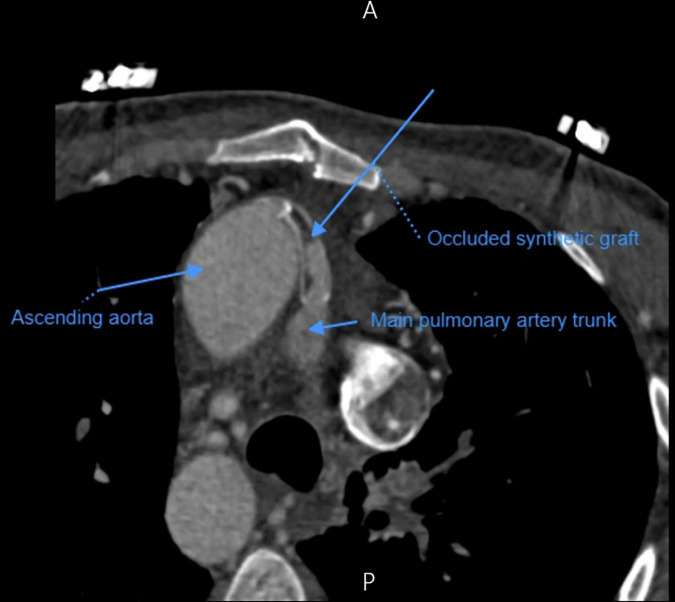
CT-scan showing occlusion of central aorto-pulmonary shunt.

**FIGURE 3:**
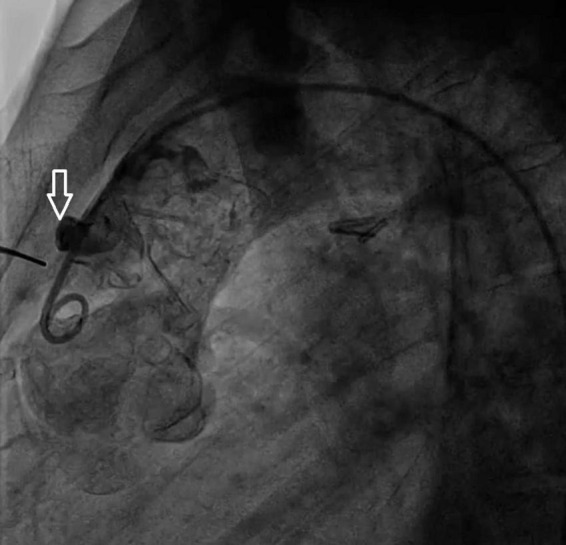
Angiographic view of the occluded shunt (arrow) before intervention.

**FIGURE 4:**
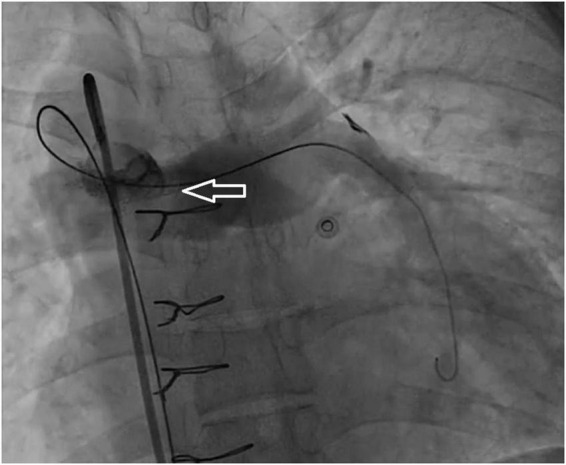
Angiographic view of the shunt (arrow) after a successful recanalization with stent placement, which shows revascularized shunt with filling of pulmonary artery vasculature.

**FIGURE 5:**
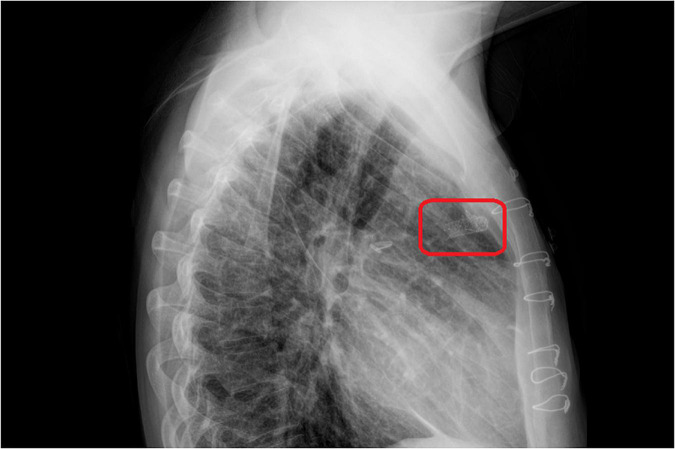
The chest X-ray on the 1 day after stent implantation. The red rectangle showed stent in the central shunt that places in a proper location without mispositioning.

A 33-year-old man was referred to our institution for progressively worsening exertional dyspnea FC III, fatigue and decreased oxygen saturation to 65% from 1 year ago. The patient had an atrial situs inversus, D-looped ventricle, double outlet right ventricle, pulmonary atresia, large ventricular septal defect, and anomalous single trunk coronary artery from ascending aorta; and has received an aorto-pulmonary central 9 mm-large Dacron shunt at the age of 25 years old. Echocardiography and CT angiography showed shunt occlusion with thrombus formation (Figure 2). Due to the high operative risk with his single right ventricle, severe ventricular enlargement, systolic dysfunction, low ejection fraction, and atrioventricular valve regurgitation, as well as anatomical difficulties, and the patient’s refusal of surgery, we proceeded with percutaneous transluminal angioplasty. From right femoral access, 7 French (F) Amplatz Left 1 guide catheter was engaged in the stump of occlusion but we were not able to cross the occlusion with a 0.035-inch hydrophilic guide wire (Radifocus; Terumo Europe, Leuven, Belgium) and V18 Control Wire (Boston Scientific, Natick, MA, USA). The angiogram showed the occluded shunt without flow (Figure 3). The obstruction was crossed using a coronary chronic total occlusion 0.014” Conquest pro (Asahi Intecc.) wire. Pre-dilation was done using 1.5 and 2.5 mm coronary balloons. The Camaro Support Catheter (QXMEDICAL) compatible with 0.035” guidewire was advanced across the lesion and the 0.014” wire was exchanged with a 0.035” Amplatz super stiff wire over which balloon dilatation was carried out using a 6 mm balloon. The systolic pulmonary artery pressure was 13 mmHg. During the first attempt to deliver a 9 mm balloon-expandable stent, the stent was dislodged and snared but inadvertently released in the descending aorta and found in the right internal iliac artery where it was kept. The stenting was successfully proceeded with another 9 mm balloon expandable stent that was delivered from left femoral artery (Figure 4). We selected the size of the balloon and balloon expandable stent based on the shunt size that was measured on the CT angiography. To ensure the appropriate placement of stent and avoid stent malposition, fluoroscopy was performed throughout the procedure and also after the intervention to confirm the location of stent. Control echocardiography and chest X-ray on the following day showed the correct stent position (Figure 5). After the procedure, the SPO2 increased to 70–75% under room air. The patient was admitted to ICU where cardioversion was needed for atrial flutter for 1 day. Due to the previous history of thrombosis, the patient was prescribed rivaroxaban with 2.5 mg dosage twice a day. The patient was discharged with an oxygen saturation of 80% and was prescribed daily aspirin and rivaroxaban. The shunt patency was closely evaluated with ultrasound assessment and confirmed on ultrasound at 1- and 3-months Post-intervention.

## Discussion

### Stent implantation for shunt failure

Maintaining the patency of SPSs play a pivotal role in the survival and quality of life of patients with shunt-dependent pulmonary circulation. Shunt failure can mostly result from shunt stenosis and occlusion, and less frequently from shunt overflow and pseudoaneurysm formation. Percutaneous stent implantation was reported in these conditions and is thereby discussed below.

#### Stent implantation for shunt stenosis or occlusion

##### Overview of shunt stenosis or occlusion

Shunt obstruction is one of the most serious complications and is associated with increased mortality and morbidity (16, 28). Shunt occlusion can occur either completely with severe hypoxemia and rapid progression to circulatory collapse or partially with progressive hypoxemia (15, 29, 30). Various mechanisms may be involved including intraluminal thrombosis and stenosis, neointimal hyperplasia, suture line fibrosis, scar formation, kinking, and vascular distortion. The formation of bacterial abscess was reported as a one rare cause of shunt stenosis (27). Most of shunts will develop over time various degrees of stenosis independently of the shunt type (31–33). Shunt obstruction can develop in two main clinical settings. Acute obstruction occurs during the first post-operative week and is often due to thrombosis. It is manifested by rapid clinical deterioration with acute hemodynamic instability, circulatory collapse, rest dyspnea, profound hypoxemia, and absence of shunt murmur. On the other side, the chronic obstruction may result from somatic growth, neointimal proliferation, peel formation, stenosis, calcification, and mural thrombus. It is characterized by progressive dyspnea and decline in oxygen saturations and exercise capacity, and progressive cyanosis leading to compensatory polycythemia (18, 22, 34). It is also noteworthy that in some patients with significant shunt narrowing around 50% are asymptomatic during the angiographic catheterization (35). Echocardiography is the most common diagnostic tool (36, 37). However, color Doppler examination of the shunt may overestimate the pulmonary arteries flow, and underestimate the degree of shunt narrowing. Therefore, diagnostic catheterization and angiography should be performed when shunt obstruction is clinically suspected (18). CT scan and MRI could delineate the shunt narrowing or occlusion, especially in cases with complex anatomies (30, 37, 38). Although the exact reason of shunt thrombosis and acute occlusion is not clearly identified, several precipitants such as shunt stenosis, supraventricular arrhythmia, perioperative shunt clampage, low weight at surgery, insufficient pulmonary artery anatomy, sepsis, and dehydration could be considered (39, 40). Longer stent length, smaller stent diameter, age at the time of surgery, and early discontinuation of anticoagulation medication are also other risk factors for compromising the shunt patency (41–44).

##### Stent implantation for shunt stenosis or occlusion

For the first time in 1997, Zahn et al. successfully exerted angioplasty and stent implantation in an occluded modified BTS in a neonate who underwent a stage 1 Norwood surgery for hypoplastic left heart syndrome and subsequently developed severe cyanosis and hemodynamic instability due to shunt thrombosis (45). In further studies, stent implantation in occluded central APS was performed in neonates and infants with good results (46,; 47). For the first time in 1999, Bader et al. reported transfemoral stenting for alleviation of shunt obstruction in 4 female patients with complex cyanotic CHD (48). Subsequently, these initial descriptions were followed by other retrospective clinical reports, with good procedural success rates and low morbidity or mortality events. Most patients experienced significant improvement in oxygen saturation and exercise tolerance upon follow-up (19, 25, 29, 48, 49). Percutaneous stent implantation may be particularly preferred in adult patients avoiding redo surgeries and the associated risks (25). Urgent shunt revascularization with drug-eluting stents has been reported in a child with thrombosed BTS with a good 9 months of follow-up on dual antiplatelet therapy (36). Also, Stent implantation following balloon angioplasty was successfully also performed in previously stented left-sided SPS that was re-obstructed with a staphylococcus aureus abscess (27).

##### Surgery for shunt stenosis or occlusion and its challenges

Urgent and definitive conventional therapy of shunt occlusion has been surgery. However, corrective surgery was not possible in most patients because of pulmonary artery hypoplasia and anatomic issues. Recently, percutaneous techniques including transcatheter thrombolysis, mechanical thrombectomy, balloon angioplasty, and stent insertion have become attractive alternatives to surgery with interesting advantages (18, 50, 51). When compared to surgery, catheter interventions offer decreased bleeding risk, post-interventional wound pain, and infection, as well as shorter hospital stay (25). In chronically compromised shunts, transcatheter restoration of shunt patency can be performed semi-electively for palliation until definitive surgery is feasible. Furthermore, these procedures can be lifesaving in patients with acute shunt occlusion and who are considered poor surgical candidates. More specifically, stent insertion may prevent or postpone re-do surgery and can provide notable clinical improvement in high-risk patients (52, 53).

##### Other treatments of shunt stenosis or occlusion and their challenges

Local administration of thrombolytic agents (urokinase or r-tPA) has been described when redo surgery was not an option, and can be effective for the treatment of acute complete shunt obstruction with an organized clot (54–56). However, several complications and adverse effects such as bleeding and failure of fibrinolytic therapy can occur and thus indicating reoperation. Moreover, fibrinolytic therapy is associated with a higher risk of bleeding in cases of early post-operative shunt thrombosis and is thereby contraindicated in those patients (54, 57–59).

Catheter-based mechanical clot disruption may be an excellent alternative therapeutic method to pharmacologic thrombolysis in cases where thrombolytic therapy is contraindicated, such as patients with recent surgery or such potentially bleeding conditions, or ineffective (60–62). The pharmacologic dissolution of thrombus to fragments using low dose r-tPA and dislodging the occlusive clot with catheter manipulation and/or balloon angioplasty could be applied *in situ* for declotting the life-threatening thrombotic shunt. In addition, thrombectomy can result in complete early recanalization and immediate improvement of shunt patency (63). However, this technique can lead to several adverse effects including fluid shifts, hemorrhage, and intravascular hemolysis. Sinus bradycardia and complete heart block could occur during and/or after intervention and have been associated with adenosine release (18). This therapeutic approach is usually ineffective in gradually developing occlusion, mural thrombus, focal neointimal hypertrophy, and calcifications. In these cases, effective graft recanalization is possible with stent implantation that will stabilize the hypertrophic neointima within the graft lumen (18, 64–66).

Previous studies reported that isolated balloon angioplasty can be an alternative to fibrinolytic therapy and redo surgery in cases with stenotic or occluded SPS offering reduced morbidity and mortality (67–70) and a variable success rate (52, 53). However, many of these patients may require an endovascular stent implantation in the future (30). Balloon dilation has the potential risk of generating neo-intimal dissection and obstructive flap (37) and the limitation of inadequately dilating the shunt with a subsequent unacceptable improvement of blood flow (53). In cases with kinking or thrombus formation, balloon dilation can be followed by residual obstruction or re-occlusion (71). The presence of fixed lesions with calcification and/or neointimal hypertrophy in the long-standing shunts notably decreases the efficacy of balloon angioplasty and requires stent placement (34, 52). Therefore, it has been suggested that percutaneous balloon angioplasty and subsequent stent implantation in obstructed SPSs could be the preferred alternative therapy, either in progressive or early postoperative shunt stenosis or occlusion (72, 73). Sent implantation should be performed in early postoperative shunt failure in which balloon dilation is contraindicated (18). Using larger balloons may be potentially associated with the risk of acute pulmonary over-circulation, however, most shunts are resistant to significant over-dilation (1).

#### Stent implantation for overflow of shunt

Like insufficient pulmonary blood flow, excessive pulmonary flow can be detrimental to patients with SPS. Pulmonary over circulation can result in pulmonary edema with reduced pulmonary compliance, decreased gas exchange and oxygen saturation, pleural effusion, systemic hypoperfusion, and consequently hemodynamic instability, especially in single-ventricle patients. So far, surgical revisions have been reported to treat medically-refractory pulmonary over circulation resulting from large SPS (74–77). Surgical approach for SPS downsizing remains the gold standard, yet it is a risky procedure in most patients. Placement of several bare-metal stents one over another inside the shunt was performed to decrease the internal shunt diameter and increase the blood flow resistance, thereby reducing the pulmonary blood flow (78). Neointimal stent proliferation might lead to an additional reduction of the lumen diameter over time (79, 80).

#### Stent implantation for pseudoaneurysm of shunt

Pseudoaneurysm formation is one of the rare but potentially fatal complications of SPSs and may be associated with shunt occlusion, infection, arterial or graft-related structural weakness, and dehiscence. The adverse events related to pseudoaneurysm may generated from rupture or compression and collapsing effect on the mediastinal structures and underlying lung parenchyma (81–85). Surgery is the traditional treatment, yet it may be associated with high morbidity and mortality, particularly in critically ill patients or those with active bleeding. In these cases, the transcatheter approach is preferred. Successful placement of covered coronary stents, after coil embolization was described in several cases with complicated large pseudoaneurysm formations (86). In addition, it has been reported that slow-growing pseudoaneurysm after BTS stent implantation was successfully excluded with a self-expandable stent graft without complication in the immediate and short-term follow-up (50).

### Vascular access for stent implantation

Stent implantation in central shunts is usually performed retrogradely from the femoral artery (45, 47). Stenting a central shunt from the radial artery was performed in adult patient (19). Other vascular accesses include the brachial and carotid arteries or the femoral vein for control angiography and stent placement especially in small infants (29). The subclavian and radial arteries were also used for re-opening without complications (25, 68, 86). Some studies reported that the carotid artery may be an alternative access to the femoral artery with a low rate of complications and a higher success rate in relieving shunt obstruction. In patients with modified BTS, carotid access provides a more direct pathway to access the shunt with less technical difficulties for the stent implantation, reduced procedural time (87–89), and no reported compromise in the carotid patency during follow-up (20).

### Stent characteristics for shunt failure

Coronary stents have been frequently used to re-open thrombotic occluded shunts, enlarge stenotic shunts, downsize large ones, and cover pseudoaneurysms (78, 86). The BTS stenting was performed with a median balloon/shunt ratio or stent/shunt ratio around 1/1 (31, 37). The diameter of implanted coronary stents was mostly 1.0–1.5 mm larger than the original shunt size. In most cases, a single stent was needed to cover the shunt length (90), yet some patients had more than one stent for proper results (35). The stent is placed across the original failed shunt under fluoroscopic guidance to avoid mispositioning (71). It has been also indicated that stents slightly larger than the original shunt size did not lead to suture line related complications (35, 37).

### Complications of stent implantation for shunt failure

Percutaneous interventions on SPSs could be associated with several complications despite no reported procedure-related death. Minor and major complications were infrequently observed and included stent malposition (29), procedural arrhythmias (i.e., atrioventricular block and junctional bradycardia), massive pericardial effusion, massive thrombo-embolic stroke, contrast media extravasation, shunt tearing (31), and acute renal failure (46, 47). Occlusive or non-occlusive arterial access thrombosis, bleeding, and hematoma may occur at the puncture site (25, 37). Femoral artery tear, spasm, and loss of a femoral pulse have been also reported (20) and the use of a long sheath was associated with a higher risk of arterial access damage (18). Several possible adverse effects including vessel rupture, dissection, vasospasm, vessel occlusion, aneurysm formation, overdilation (91–93), and pseudoaneurysm are possible complications of stent insertion (50). Pulmonary hemorrhage caused by a tear of the pulmonary artery and occlusion of the distal pulmonary artery were also reported (30). Dilation and stenting of thrombotic shunts might lead to the embolization of thrombotic material into the pulmonary arteries (71). Stent thrombosis, even despite anti-platelet therapy, and stent stenosis secondary to intimal growth are possible short and long-term complications of stented shunts (25). Bonnet et al. reported in a large series no statistical association between complications and age, weight, or number of stents placed (31).

### Prognosis of stent implantation for shunt failure

Stenting obstructed SPSs are usually successful procedures with low morbidity rates (15, 23, 48). One large study showed that 93% of the interventions were successful (20). Another study reported an 84.8% early procedural success rate of which 78.8% remained long-lasting. The failure rate of isolated angioplasties was higher than the one with concomitant stenting (31). Another large series reporting on transcatheter stenting in various groups of patients with SPS showed a success rate of 92.3%. This study confirmed the effectiveness and safety of stent placement to treat acute post-operative shunt obstruction, inter-stage shunt dysfunction, and chronic shunt stenosis, thereby avoiding surgical re-intervention. Additionally, this study demonstrated that only one out of seven failed cases received stent insertion, while the other 6 cases underwent angioplasty without stenting (35). Evidence of restenosis can be found on different imaging modalities assessments several years after the first intervention and may need re-stenting (19, 66). Another study reported that transcatheter procedures in 23 BTSs were successful and effective in 96% of cases (94). Another study reported that stent implantation could prolong the lifespan of a surgically inserted SPS. Nonetheless, stenosis secondary to intimal proliferation can be described, particularly in those who had SPS for a long time. In this study, 20% patients returned to the catheterization laboratory for angioplasty or additional stent implantation (35). Previous reports have demonstrated that stent insertion within SPSs does not interfere with the later surgeries, and no technical difficulties were observed in surgically removed stented shunts (35, 71). Overall, despite some major intervention-related complications, catheter-based procedures with stenting can be considered as a less invasive approach, compared with re-do surgery, to restore the efficacy and patency of SPSs in critically ill patients with an acceptable success rate.

### Anticoagulant therapy after stent implantation

Anticoagulant therapy could be considered in cases with thrombosis or in subjects at high risk for thrombotic events. Heparin was given before stenting for all patients with close monitoring of activated clotting time around 200 s (66, 95). Post-procedure, continuous intravenous heparin infusion was given and was followed by oral aspirin (96). During the intubation and intensive care unit (ICU) admission, low dose heparin infusion could be used until patients can tolerate oral intake (35). Patients could be discharged on single or combined anticoagulant therapy of low molecular heparin, aspirin (97), warfarin (with a target INR of between 2.5 and 3.5) (48), or clopidogrel (50), usually for a total of 3–6 months (29). Prasugrel can be administered in patients with repetitive shunt thrombosis (31). Dual antiplatelet therapy with aspirin and clopidogrel may be used with drug-eluting stents (36). Additionally, dual antiplatelet therapy could be considered to protect the shunt in patients at high risk of thrombosis. In cases with simultaneous peripheral arterial thrombus, Lovenox could be considered in addition to aspirin (18, 35).

### Follow-up of patients after stent implantation

#### Pediatric patients (<18 years)

The follow-up length after stent insertion in failed SPSs reported in the literature varied from months to years (33, 36, 58). Most studies reported on followed of patients within 3–6 months after procedure by measuring oxygen saturation (53, 66, 78, 96), and by catheterization (45), or echocardiography (99) or angiography (66) to evaluate the shunt patency.

Neonates and infants receiving shunt stenting subsequently underwent surgery within a few months after stent placement, either for complete repair or for staged palliative repair (37, 71, 96, 97). Palliative or corrective surgery could be delayed for more than 1 year with no reported deaths or episodes of stent thrombosis during follow-up (86, 90). This delay can go up to a median of 8.8 years as reported by Bonnet et al. (31). Another study showed that shunts downgraded in size with endovascular stenting were all patent at a median follow-up of 7.3 months with no signs or symptoms of pulmonary over-circulation (95). Also, no procedure-related mortality was reported during follow-up period (71, 90, 96).

#### Adult patients (>18 years)

The available data on stenting of SPSs in adults is limited. The length of the follow-up reported in published series ranged from a few months to years (50). One study reported no complication on a follow-up of 2 months after stenting (53). Bader et al. reported a longer follow-up (range 1.6–3.5 years) of four patients of which two patients remained asymptomatic with patent shunts, one patient with stented APS, received a modified BTS distal to the migrated stent, and one patient non-compliant to his treatment thrombosed his stented shunt 6 months after the procedure and was successfully treated with warfarin (48). Another study showed that patients were followed with physical examination, laboratory data, and measuring saturation. Echocardiography was used to evaluate the shunt patency and found no complication (25). MRI imaging can also be useful to detect shunt/stent narrowing in asymptomatic patients during follow-up (19).

Altogether, there is a controversy regarding the follow-up of patients who underwent stent implantation in SPSs. It appears to be reasonable to follow these patients 1 month after the procedure and thereafter every 3 months by physical examination, oxygen saturation measurement, and echocardiography for assessing shunt patency. In cases with suspected shunt failure, CT-angiography is helpful.

## Conclusion and future prospectives

Percutaneous treatment of failed SPSs, particularly stenotic or occluded ones, is an appealing alternative in selected adult and pediatric patients who are not good candidates for redo surgeries. Transcatheter strategies, including transcatheter pharmacologic thrombolysis, mechanical thrombectomy, balloon angioplasty, and stent implantation, can be applied with reasonable safety and significant clinical efficacy. Despite limited evidence in the literature, it appears that shunt stenting is a more attractive alternative to surgery and the other transcatheter options. The lack of information and guidelines on the follow-up of these challenging patients after the stenting procedure is another additional challenge to the clinician and the interventionist. In this regard, large scale long-term multi-centric studies are needed to determine the efficacy of stent implantation for SPS failure in the long run.

## Author contributions

YJ, KH, and HG: conception and design. YJ and HG: intervention and patient clinical follow-up patient. MR: drafting of the manuscript. YJ, KH, HG, RH, and AZ: revising manuscript critically for important intellectual content. MR and HG: final approval of the manuscript submitted. All authors have read and approved the submitted manuscript.

## Abbreviations

APS: aortopulmonary shunt
BTS: Blalock-Taussig shunt
CHD: congenital heart defect
CT scan: computed tomography scan
MRI: magnetic resonance imaging
r-tPA: recombinant tissue plasminogen activator
SPS: systemic-to-pulmonary arterial shunt.
