# Corrigendum: Percutaneous stent implantation for occluded central shunts in adults: A case report and review of current evidence

**DOI:** 10.3389/fcvm.2022.1106524

**Published:** 2023-01-05

**Authors:** Yaser Jenab, Malihe Rezaee, Kaveh Hosseini, Homa Ghaderian, Raymond N. Haddad, Ali N. Zaidi

**Affiliations:** ^1^Tehran Heart Center, Cardiovascular Diseases Research Institute, Tehran University of Medical Sciences, Tehran, Iran; ^2^School of Medicine, Shahid Beheshti University of Medical Sciences, Tehran, Iran; ^3^M3C-Necker, Hôpital Universitaire Necker-Enfants Malades, Assistance Publique-Hôpitaux de Paris (AP-HP), Paris, France; ^4^Mount Sinai Adult Congenital Heart Disease Center, Icahn School of Medicine, New York, NY, United States

**Keywords:** aortopulmonary shunt, systemic-to-pulmonary shunt, stent, percutaneous intervention, central shunt

In the published article, there were some errors.

A correction has been made to the **Abstract**. This sentence previously stated:

“We also reported the case of a 33-year-old man with cyanotic CHD and a occluded central aorto-pulmonary shunt”

The corrected sentence appears below:

“We also reported the case of a 33-year-old man with cyanotic CHD and an occluded central aorto-pulmonary shunt”

A correction has been made to the **Introduction**, paragraph one. This sentence previously stated:

“Despite advancements in corrective surgeries for congenital heart defects (CHDs), systemic-to-pulmonary arterial shunts (SPSs) remain the most common in complex CHDs with inadequate pulmonary blood flow”

The corrected sentence appears below:

“Despite advancements in corrective surgeries for congenital heart defects (CHDs), systemic-to-pulmonary arterial shunts (SPSs) remain the most common surgical palliation in complex CHDs with inadequate pulmonary blood flow”

A correction has been made to the **Results** section. This sentence previously stated:

“108 (72%) patients had classical or modified BTS, 37 (24.7%) patients had APS, 3 (2%) patients had both BTS and APS, one patient had a left-sided systemic-to-pulmonary shunt, and one patient had a RIMA to pulmonary artery shunt.”

The corrected sentence appears below:

“108 (72%) patients had classical or modified BTS, 37 (24.7%) patients had APS, 3 (2%) patients had both BTS and APS, one patient had a left-sided SPS, and one patient had a right internal mammary artery (RIMA) to pulmonary artery shunt.”

A correction has been made to the **Results**. This sentence previously stated:

“Shunt stenosis and thrombosis was the most indication for endovascular stent implantation and patients mainly presented with cyanosis and decreased atrial oxygen saturations.”

The corrected sentence appears below:

“Shunt stenosis and thrombosis were the most indications for endovascular stent implantation and patients mainly presented with cyanosis and decreased atrial oxygen saturation.”

A correction has been made to the **Results**. This sentence previously stated:

“One patient with a history of percutaneously treated stenotic shunt underwent 5 years later stent implantation in a left-sided SPS after a bacterial abscess induced shunt obstruction.”

The corrected sentence appears below:

“One patient with a history of percutaneously treated stenotic shunt underwent after 5 years a stent implantation in a left-sided SPS after a bacterial abscess induced shunt obstruction.”

A correction has been made to the **Brief case presentation**, paragraph one. This sentence previously stated:

“and was received an aorto-pulmonary central 9 mm-large Dacron shunt at the age of 25 years old. Echocardiography and CT angiography showed shunt occlusion with thrombus”

The corrected sentence appears below:

“and has received an aorto-pulmonary central 9 mm-large Dacron shunt at the age of 25 years old. Echocardiography and CT angiography showed shunt occlusion with thrombus formation”

A correction has been made to the **Brief case presentation**, paragraph one. This sentence previously stated:

“Due to the high operative risk because of single ventricle that was right ventricle, sever enlargement of ventricle, systolic dysfunction, low ejection fraction, and atrioventricular”

The corrected sentence appears below:

“Due to the high operative risk with his single right ventricle, severe ventricular enlargement, systolic dysfunction, low ejection fraction, and atrioventricular”

A correction has been made to the **Brief case presentation** section. The sentence previously stated:

“After procedure SPO2 increased to 70–75% under room air. The patient was admitted to ICU where cardioversion was needed for atrial flutter for 1 day. Due to the previous history of thrombosis, the patient was prescribed rivaroxaban with 2.5 mg dosage twice a day. The patient was discharged with an oxygen saturation of 80% and was prescribed daily aspirin and rivaroxaban. The shunt patency was closely evaluated with ultrasound assessment and is confirmed at 4 months of follow-up, by echocardiography 1 month after discharge, thereafter 3 months later.”

The corrected sentence appears below:

“After the procedure, the SPO2 increased to 70–75% under room air. The patient was admitted to ICU where cardioversion was needed for atrial flutter for 1 day. Due to the previous history of thrombosis, the patient was prescribed rivaroxaban with 2.5 mg dosage twice a day. The patient was discharged with an oxygen saturation of 80% and was prescribed daily aspirin and rivaroxaban. The shunt patency was closely evaluated with ultrasound assessment and confirmed on ultrasound at 1- and 3-months Post-intervention.”

A correction has been made to the **Discussion**, subsection “*Stent implantation for shunt stenosis or occlusion*,” subsection “*Overview of shunt stenosis or occlusion*.” This sentence previously stated:

“Longer stent length, smaller stent diameter, age at the time of surgery, and early discontinuation of anticoagulation medication are also other risk factors for shunt patency”

The corrected sentence appears below:

“Longer stent length, smaller stent diameter, age at the time of surgery, and early discontinuation of anticoagulation medication are also other risk factors for compromising the shunt patency”

A correction has been made to the **Discussion**, subsection “*Stent implantation for shunt stenosis or occlusion*,” subsection “*Stent implantation for shunt stenosis or occlusion*.” The sentence previously stated:

“For the first time in, Zahn et al. successfully exerted angioplasty and stent implantation in an occluded modified BTS in a neonate who underwent a stage 1 Norwood surgery for hypoplastic left heart syndrome and subsequently developed severe cyanosis and hemodynamic instability due to shunt thrombosis (45). In further studies in, stent implantation in occluded central APS was performed in neonates and infants with good results (46, 47). For the first time in, Bader et al. reported transfemoral stenting for alleviation of shunt obstruction in 4 female patients with complex cyanotic heart disease”

The corrected sentence appears below:

“For the first time in 1997, Zahn et al. successfully exerted angioplasty and stent implantation in an occluded modified BTS in a neonate who underwent a stage 1 Norwood surgery for hypoplastic left heart syndrome and subsequently developed severe cyanosis and hemodynamic instability due to shunt thrombosis (45). In further studies, stent implantation in occluded central APS was performed in neonates and infants with good results (46, 47). For the first time in 1999, Bader et al. reported transfemoral stenting for alleviation of shunt obstruction in 4 female patients with complex cyanotic CHD.”

A correction has been made to the **Discussion**, subsection “*Stent implantation for shunt stenosis or occlusion*,” subsection “*Other treatments of shunt stenosis or occlusion and their challenges*,” paragraph one. This sentence previously stated:

“However, several complications and adverse effects such as bleeding and failure of fibrinolytic therapy can occur and thus indicating reoperation. Moreover, fibrinolytic is associated with a higher risk of bleeding in cases of early post-operative shunt thrombosis and is thereby contraindicated in those patients”

The corrected sentence appears below:

“However, several complications and adverse effects such as bleeding and failure of fibrinolytic therapy can occur and thus indicating reoperation. Moreover, fibrinolytic therapy is associated with a higher risk of bleeding in cases of early post-operative shunt thrombosis and is thereby contraindicated in those patients”

A correction has been made to the **Discussion**, subsection “*Complications of stent implantation for shunt failure*.” This sentence previously stated:

“Percutaneous interventions on SPSs could be r despite no reported procedure-related death.”

The corrected sentence appears below:

“Percutaneous interventions on SPSs could be associated with several complications despite no reported procedure-related death.”

A correction has been made to the **Discussion**, subsection “*Prognosis of stent implantation for shunt failure*,” paragraph one. This sentence previously stated:

“One large study showed that 93% of the interventions on were successful”

The corrected sentence appears below:

“One large study showed that 93% of the interventions were successful”

A correction has been made to the **Discussion**, subsection “*Anticoagulant therapy after stent implantation*.” This sentence previously stated:

“Anticoagulant therapy could be considered in cases with thrombotic or in subjects at high risk for thrombotic events.”

The corrected sentence appears below:

“Anticoagulant therapy could be considered in cases with thrombosis or in subjects at high risk for thrombotic events.”

A correction has been made to the **Discussion**, subsection “*Prognosis of stent implantation for shunt failure*.” This sentence previously stated:

“not interfere with the later surgeries, and no technical difficulties were observed in surgically removing stented shunts”

The corrected sentence appears below:

“not interfere with the later surgeries, and no technical difficulties were observed in surgically removed stented shunts”

A correction has been made to the **Discussion**, subsection “*Follow-up of patients after stent implantation*,” subsection “*Pediatric patients (*<*18 years)*,” paragraph one. This sentence previously stated:

“The follow-up length after stent insertion in failed SPSs reported in the literatures varied from months to years (31, 34, 52). Most studies reported followed of patients within 3–6 months after procedure by measuring oxygen saturation (53, 66, 78, 96), and by catheterization (45), or echocardiography (98) or angiography (66) to evaluate the shunt patency.”

The corrected sentence appears below:

“The follow-up length after stent insertion in failed SPSs reported in the literature varied from months to years (33, 36, 58). Most studies reported on followed of patients within 3–6 months after procedure by measuring oxygen saturation (53, 66, 78, 96), and by catheterization (45), or echocardiography (99) or angiography (66) to evaluate the shunt patency.”

A correction has been made to the **Discussion**, subsection “*Follow-up of patients after stent implantation*,” subsection “*Pediatric patients (*<*18 years)*,” paragraph two. This sentence previously stated:

“Palliative or corrective surgery could be thereby delayed for more than 1 year with no reported deaths or episodes of stent thrombosis during follow-up”

The corrected sentence appears below:

“Palliative or corrective surgery could be delayed for more than 1 year with no reported deaths or episodes of stent thrombosis during follow-up”

A correction has been made to the **Discussion**, subsection “*Follow-up of patients after stent implantation*,” subsection “*Adult patients (*>*18 years)*,” paragraph one. This sentence previously stated:

“and one patient non-compliant to his treatment experienced thrombosed stented shunt 6 months after the procedure and was successfully treated with warfarin”

The corrected sentence appears below:

“and one patient non-compliant to his treatment thrombosed his stented shunt 6 months after the procedure and was successfully treated with warfarin”

The authors apologize for these errors and state that these do not change the scientific conclusions of the article in any way. The original article has been updated.

