# Supplementary material for: Percutaneous stent implantation for occluded central shunts in adults: A case report and review of current evidence
Source: Front Cardiovasc Med. 2022 Nov 21;9:1032974. doi: 10.3389/fcvm.2022.1032974 (PMC9720738; doi:10.3389/fcvm.2022.1032974)
Supplement: Supplementary file 1 [file Table_1.DOCX]

| **Table 1. Characteristics of the studies** | | | | | | | | | | | | |
| --- | --- | --- | --- | --- | --- | --- | --- | --- | --- | --- | --- | --- |
| References | Year of publication | Type of study | Number of patients | Age at shunt creation | Sex | Congenital heart disease | Type of shunt | Age at stenting | Time from shunt to stent | Indication of stent implantation | Symptom before stenting | Outcome |
| (1) | 1997 | Case report | 1 | 8 d | NR | HLHS | mBTS | Neonate | ------ | Shunt thrombosis and stenosis | Progressively cyanosis  Dropped SpO2  Hypotension | Successful |
| (2) | 1998 | Case series | 2 | 10 d | NR | TA | BTS | 15 d | 5d | Thrombotic shunt occlusion | Hypoxemia  Dropped SpO2 | Successful |
|  |  |  |  | 4 W | NR | TOF with hypoplastic pulmonary arteries | BTS | 6 W | 2w |  |  |  |
| (3)  Not English  (Based on abstract) | 1999 | Case report | 2 | ------ | ------ | Complex cyanotic CHD | Central APS | 1 m and 13 m | -------- | Shunt occluded | Severe hypoxemia | Successful |
| (4)  Full text not found  (Based on abstract) | 1999 | Case report | 1 | ---- | ---- | TA | Central APS | 5.5 W | -------- | Severe postoperative stenosis | Severe hypoxia | Successful |
| (5) | 1999 | Case series | 4 | 5 – 29 y | F | Complex cyanotic CHD | 1 BTS  2 mBTS  1 central APS | 23 y – 32 y | 2 – 21 y | Long segment stenosis of shunts | Increasing cyanosis  Decrease SpO2 | Successful |
| (6)  In Spanish  (Based on abstract) | 1999 | Case report | 1 | In infancy | F | PA/VSD | Classic BTS | 26 y | ------- | Complete obstruction | Progressive cyanosis  Dyspnea | Successful |
| (7) | 2000 | Case series | 2  (2 of 3 patients underwent stent in shunt) | 28 y | F | PA/VSD | BTS | 33 y | 5 y | A discrete stenosis | Increasing cyanosis  Dropped SpO2  Hypoxemia | Successful |
|  |  |  |  | 8 y | M | PA/VSD | mBTS | 11 y | 3 y | Severe shunt narrowing |  |  |
| (8) | 2001 | Case series | 13  (15 stent implantations) | 1 d - 8 y | 8 M  5 F | Complex cyanotic CHD | 2 classic BTS  10 mBTS  1 central APS | 14 d - 12.5 y | Median: 1.4y (1 d - 8 y) | Shunt stenosis or occlusion | Low arterial SpO2 Change in the shunt murmur | Successful |
| (9) | 2002 | Case report | 1 | 1 m | M | PA | mBTS | 3 y | 2 y & 11 m | Acute obstruction following selective angiography | Decrease the SpO2 | Successful |
| (10)  In polish (Based on abstract) | 2004 | Case report | 1 | ----- | M | Complex cyanotic CHD | mBTS | 5 y | ---- | ------- | ---------- | Successful |
| (11) | 2005 | Case report | 1 | 15 d | F | Complex cyanotic CHD | Classical BTS | 10 y | 10 y | Severe stenosed shunt | Severe cyanosis  Decreased SpO2 Severe limitation in physical activity | Successful |
| (12)  Full text not found | 2006 | Case report | 1 | 4 y | F | Complex cyanotic CHD | mBTS | 21 y | 17 y | Narrowed shunt with impaired blood flow | Declining exercise tolerance  Presyncope  Increasing cyanosis | successful |
| (13) | 2007 | Case report | 1 | 3 d | M | HLHS | mBTS | 50 d | 47 d | shunt stenosis | SPO2 dropped  Quieter shunt murmur | Successful |
| (14) | 2008 | Case series | 7 | NR | NR | Complex cyanotic CHD | 6 central APS  1 mBTS | 6 d - 7 m | ------ | Acute shunt occlusion | Acute decreased SpO2 | Successful |
| (15) | 2008 | Case series | 3  (3 of 5 patients have SPS) | NR | NR | Complex cyanotic CHD | 2 central APS  1 mBTS | 12 d - 62 d | ------ | Partial or complete occluded shunt | Decreased SpO2 | Successful |
| (16) | 2010 | Case series | 3  (3 of 23 have stent implantations) | NR | M | Complex cyanotic CHD | mBTS | 18 d | 5 d | Occlusion or stenosis of shunts | Decreased SpO2 | 3 successful |
|  |  |  |  |  |  |  |  | 2 y | 3 y |  |  |  |
|  |  |  |  |  |  |  |  | 5 y | 8 m |  |  |  |
| (17) | 2010 | Case report | 1 | BTS inserted in 4 years old    Central APS inserted in 6 years old | F | PA/VSD | Classic BTS and central APS  (Just stenting in BTS) | 26 y | 22 y | Complete obstruction of the BTS | Progressive cyanosis Dyspnea | Successful |
|  |  |  |  |  |  |  |  | 40 y | 14 y | Pseudoaneurysm- with a dissection flap inside immediately proximal to the stent | In CT and CXR | Successful |
| (18) | 2011 | Case series | 7 | NR | 6 M  1 F | Complex cyanotic CHD | 2 Classical/ 5 modified BTS | 25 d - 218 d | 2 d - 137 d | Shunt stenosis  or occlusion | Low SpO2  Tachypnoea | 7 Successful |
| (19) | 2012 | Case report | 1 | 1 w | M | Complex cyanotic CHD with PA | Bilateral mBTS | 5.75 y | 5 y | Severe stenosis of both BT shunts | Decrease SpO2 at rest and in sleep  Cyanosis of lip  Clubbing  Quieter shunt murmur | Successful |
| (20) | 2013 | Case report | 1 | 4 m | M | Complex cyanotic CHD with PA | Central APS | 16 m | 12 m | Shunt stenosis | Decreased SpO2 | Successful |
| (21) | 2015 | Case series | 14  (14 of 28 have stent implantations) | 0.01 y –18 y | M/F sex ratio = 1.15 | PS/ PA only or with another defects | mBTS | 0.03y –32 y with  (18 % of adults (>15y)) | ------ | Shunt occlusion | Decrease SpO2  Change in murmur  Dyspnea | 13 successful  1 unsuccessful |
| (22) | 2015 | Case series | 22 (25 stent implantation) | NR | 9 F  13 M | Complex cyanotic CHD with PA | 13 mBTS  7 central APS  1 RIMA to pulmonary artery shunt  1 both central and BTS | 10 d - 4 y | Median 1.9 m (4 d –3.8y) | Shunt occlusion or narrowing | Progressive cyanosis  Oxygen requirement  Respiratory distress  Increasing cyanosis  Cardiovascular collapse | 25 Successful |
| (23) | 2016 | Case report | 1 | 7 y | F | Complex cyanotic CHD | BTS | 16 y | 9 y | Oversized shunt | Massive left sided pleural effusions with hemodynamic compromise | Successful |
| (24) | 2016 | Case report | 2 | 3 y and 10 m | F | DOVR with PS | mBTS | 4 y | 2 m | Pseudoaneurysm of shunt | Intermittent massive pulmonary hemorrhage  Decrease SpO2  Enlarged upper left mediastinum | Successful |
|  |  |  |  | 7 y | M | Complex cyanotic CHD | mBTS | 7 y and 5 m | 5 m | Pseudoaneurysm of shunt | Massive hemoptysis | Successful |
| (25) | 2017 | Case series | 11  (11 of 19 patients, need stent) | 0.2 m – 16 m | NR | Complex cyanotic CHD | Central APS | 2 m -20 m | Median: 3 m (0.9-4.4m) | Shunt stenosis | Decrease SpO2 | 11 successful |
| (26) | 2017 | Case series | 34 patients (42 stent insertion) | NR | NR | Complex cyanotic CHD | BTS | 8 - 1,634 d | ------- | Stenotic shunt or occlusion | Cyanosis | 39 of 42 stents (93%) successful  3 of 49 stents unsuccessful |
| (27) | 2018 | Case series | 5 | NR | 4 F  1 M | Complex cyanotic CHD with PA | 1 Classical BTS  2 mBTS  2 Central APS | 17 y – 45 y | ------ | Stenotic shunt or occlusion | Decrease SpO2  Progressive cyanosis | 5 Successful |
| (28) | 2019 | Case report | 1 | 7 y | M | PA/VSD | mBTS and  central APS  (Stenting just in mBTS) | 47 y | 20 y | Occluded BT shunt | Progressive fatigue  Limited walking distance  Hypoxemia  Decreased SPO2 | Successful |
| (29) | 2020 | Case series | 4 | NR | NR | Complex cyanotic CHD | 3 central APS  1 mBTS | 0.7 y –5.7 y | median: 56 d (26 – 160 d) | Oversized shunt | Signs or symptoms of pulmonary over circulation | 4 Successful |
| (30) | 2020 | Case report | 1 | 7 m | M | DOVR/PS | BTS | 7 m | 10 d | Acute shunt thrombosis | Decrease SPO2  Shock  Absence the Shunt murmur | Successful |
| (31) | 2020 | Case report | 1 | 1 m | F | Single ventricle/ PA/ arterial-pulmonary collateral arteries | left-sided systemic-to-pulmonary shunt | 8 y | 8 y | Shunt stenosis | Decrease SPO2  Hemodynamic instability | successful |
|  |  |  |  |  |  |  |  | 13 y | 13 y | Shunt obstruction due to a Staphylococcus aureus abscess |  |  |

**Reference:**

1. Zahn EM, Chang AC, Aldousany A, Burke RP. Emergent stent placement for acute Blalock-Taussig shunt obstruction after stage 1 Norwood surgery. Catheterization and cardiovascular diagnosis 1997;42(2):191-4.

2. Peuster M, Fink C, Bertram H, Paul T, Hausdorf G. Transcatheter recanalization and subsequent stent implantation for the treatment of early postoperative thrombosis of modified Blalock-Taussig shunts in two children. Catheterization and cardiovascular diagnosis. 1998;45(4):405-8.

3. Alcíbar J, Peña N, Cabrera A, Jiménez A, Gómez S, de La Torre J, et al. Stent implantation in palliative central aortopulmonary shunt of congenital cardiopathies with pulmonary hypoperfusion. Experience of 2 cases. Revista Espanola De Cardiologia. 1999;52(10):863-8.

4. Alcibar J, Cabrera A, Martínez P, Peña N, Oñate A. Stent implantation in a central aorto-pulmonary shunt. The Journal of Invasive Cardiology. 1999;11(8):506-9.

5. Bader R, Somerville J, Redington A. Use of self expanding stents in stenotic aortopulmonary shunts in adults with complex cyanotic heart disease. Heart. 1999;82(1):27-9.

6. Benito Bartolomé F, Sánchez Fernández-Bernal C, Garzón Mol G, Oliver Ruiz J. [Implantation of stents in Blalock-Taussig shunt in an adult patient with pulmonary atresia and interventricular septal defect]. Rev Esp Cardiol. 1999;52(9):730-2.

7. El-Said HG, Clapp S, Fagan TE, Conwell J, Nihill MR. Stenting of stenosed aortopulmonary collaterals and shunts for palliation of pulmonary atresia/ventricular septal defect. Catheterization and cardiovascular interventions. 2000;49(4):430-6.

8. Lee K-J, Humpl T, Hashmi A, Nykanen DG, Williams WG, Benson LN. Restoration of aortopulmonary shunt patency. American Journal of Cardiology. 2001;88(3):325-8.

9. Tomita H, Hayashi G, Echigo S. "Bail-out" stenting for acute obstruction of a modified Blalock-Taussig shunt following selective angiography. Cardiology in the Young. 2002;12(5):496-8.

10. Moszura T, Ostrowska K, Dryżek P, Moll J, Sysa A. Thrombolysis and stent implantation in a child with an acute occlusion of the modified Blalock-Taussig shunt-a case report. Kardiologia Polska (Polish Heart Journal). 2004;60(4):354-6.

11. Kouatli A, Al-Ata J, Galal MO, Amin MA, Hussain A. Stent implantation to maintain patency of a stenosed Blalock Taussig shunt. Asian Cardiovasc Thorac Ann. 2005;13(3):274-6.

12. Maree A, Walsh K. Coronary stent insertion into a 20-year-old Blalock-Taussig Shunt. Irish Medical Journal. 2006;99(7):218-.

13. Krasemann T, Qureshi S. Stenting of a stenosed modified Blalock Taussig shunt after Norwood-I palliation for hypoplastic left heart. Heart. 2007;93(12):1509.

14. Sreeram N, Emmel M, Ben-Mime L, Brockmeier K, Bennink G. Transcatheter recanalization of acutely occluded modified systemic to pulmonary artery shunts in infancy. Clin Res Cardiol. 2008;97(3):181-6.

15. Kaestner M, Handke RP, Photiadis J, Sigler M, Schneider MB. Implantation of stents as an alternative to reoperation in neonates and infants with acute complications after surgical creation of a systemic-to-pulmonary arterial shunt. Cardiol Young. 2008;18(2):177-84.

16. Moszura T, Zubrzycka M, Michalak KW, Rewers B, Dryżek P, Moll JJ, et al. Acute and late obstruction of a modified Blalock–Taussig shunt: a two-center experience in different catheter-based methods of treatment. Interactive cardiovascular and thoracic surgery. 2010;10(5):727-31.

17. Sanchez-Recalde A, Garzón G, Oliver JM. Stent graft exclusion of a pseudoaneurysm in a Blalock-Taussig shunt. Catheter Cardiovasc Interv. 2010;76(2):251-6.

18. Krasemann T, Tzifa A, Rosenthal E, Qureshi SA. Stenting of modified and classical Blalock–Taussig shunts–lessons learned from seven consecutive cases. Cardiology in the Young. 2011;21(4):430-5.

19. Lee M-L, Chiu S. Stent implantation for stenotic Blalock–Taussig shunts in a 5.75-year-old boy with pulmonary atresia. International Journal of Cardiology. 2012;162(1):e8-e11.

20. McMahon CJ, Franklin O, Walsh KP. Stenting of central aortopulmonary shunt in a child with pulmonary atresia, ventricular septal defect, and severely hypoplastic pulmonary arteries. Congenital Heart Disease. 2013.

21. Bonnet M, Petit J, Lambert V, Brenot P, Riou J-Y, Angel C-Y, et al. Catheter-based interventions for modified Blalock–Taussig shunt obstruction: a 20-year experience. Pediatric Cardiology. 2015;36(4):835-41.

22. Vaughn GR, Moore JW, Mallula KK, Lamberti JJ, El‐Said HG. Transcatheter stenting of the systemic‐to‐pulmonary artery shunt: A 7‐year experience from a single tertiary center. Catheterization and Cardiovascular Interventions. 2015;86(3):454-62.

23. Fiszer R, Szkutnik M, Iashchuk N, Bialkowski J. A case of percutaneous modified Blalock-Taussig shunt downsize with multiple stent-in-graft technique. Postępy w Kardiologii Interwencyjnej= Advances in Interventional Cardiology. 2016;12(2):164.

24. Baspinar O, Sahin DA, Sulu A, Gokaslan G. Interventions Involving the Use of Covered Coronary Artery Stents for Pseudoaneurysms of Blalock-Taussig Shunts. World J Pediatr Congenit Heart Surg. 2016;7(4):494-7.

25. Cools B, Brown SC, Boshoff DE, Eyskens B, Heying R, Rega F, et al. Percutaneous intervention for central shunts: new routes, new strategies. Acta Cardiologica. 2017;72(2):142-8.

26. Ligon RA, Ooi YK, Kim DW, Vincent RN, Petit CJ. Intervention on surgical systemic-to-pulmonary artery shunts: carotid versus femoral access. JACC: Cardiovascular Interventions. 2017;10(17):1738-44.

27. Kasem M, Bentham J, Thomson J. Single-centre experience in stenting arterial shunts for adult CHD patients with single-ventricle physiology and pulmonary blood flow dependent on arterial shunts. Cardiology in the Young. 2018;28(12):1431-5.

28. Illner J, Reinecke H, Baumgartner H, Kaleschke G. Stenting of modified Blalock–Taussig shunt in adult with palliated pulmonary atresia and ventricular septal defect: a case report. European Heart Journal: Case Reports. 2019;3(4):1.

29. Maschietto N, Baird C, Porras D. Percutaneous intraluminal downsizing of systemic‐to‐pulmonary artery shunts: a novel application of the Diabolo stent technique—Case series and description of the technique. Catheterization and Cardiovascular Interventions. 2020;95(3):471-6.

30. Gopalakrishnan A, Sasidharan B, Menon S, Krishnamoorthy KM. Drug-eluting stent for acute Blalock-Taussig shunt thrombosis in a child-case report. Egypt Heart J. 2020;72(1):54.

31. Homma Y, Hayabuchi Y. Successful treatment by stent implantation for systemic-to-pulmonary shunt obstruction due to a Staphylococcus aureus abscess: a case report. Cardiology in the Young. 2020;30(10):1538-40.
